# The biological state hypothesis: biological state as a systems-level constraint on human adaptive capacity

**DOI:** 10.3389/fphys.2026.1909109

**Published:** 2026-07-30

**Authors:** Aly Gallo López

**Affiliations:** Center for Research in Precision Medicine, Genomics, and Human Performance, Lima, Peru

**Keywords:** adaptive capacity, adaptive reserve, biological plasticity, biological state, chronic non-communicable disease, integrated physiological coherence, systemic constraint, systems physiology

## Abstract

Contemporary biomedicine has largely approached the human organism in a fragmented way: each specialty addresses its own system, each biomarker its own domain. This reductionist model has produced precise knowledge of parts, yet remains poorly suited to explain the systemic nature of chronic disease and accelerated biological aging—and, in particular, why individuals exposed to comparable biological and environmental demands follow radically different adaptive trajectories, with some losing their capacity to respond long before any diagnosable pathology appears. We propose the biological state hypothesis (BSH), a theoretical framework in systems physiology. Biological state is defined as the dynamic expression of the functional order with which the organism engages its environment—an integrated physiological condition emerging from the continuous interaction among three interdependent layers: systemic regulation (the coordinated activity of the autonomic, neuroendocrine, immune-inflammatory, and circadian axes), metabolism (the capacity to generate, allocate, and utilize energy for regulatory, repair, and adaptive processes), and biological plasticity (neuroplasticity and epigenetic–genomic plasticity, through which sustained environmental pressures are inscribed into the organism’s biological architecture). Environmental effects on biological plasticity occur predominantly—although not exclusively—through systemic regulation and metabolism, which together condition how the organism’s architecture is remodeled. The central proposition is that adaptive capacity—the organism’s capacity to maintain or restore integrated physiological coherence when confronted with environmental demands—is an emergent functional property of integrated biological state. When the three layers maintain coherence, adaptive reserve is broad; when dysregulation persists across domains, it progressively narrows. Within this framework, chronic non-communicable disease and accelerated biological aging are understood not as primary events but as downstream consequences of a sustained deterioration of biological state—one that first manifests as a narrowing of adaptive capacity and only later as overt pathology. Presented as a falsifiable framework rooted in the cross-domain integration of multiple physiological domains, the BSH generates five empirically testable predictions and provides a conceptual foundation for understanding, measuring, and restoring human adaptive capacity across the life course.

## 1 Introduction

### 1.1 What determines how much adaptive capacity remains available to the organism at any given moment—and in which direction is that capacity trending?

The central question of this hypothesis is not how much damage an organism has accumulated, but how much physiological capacity it retains to respond, recover, and adapt in the face of future demands.

This question is the starting point of the biological state hypothesis. Interindividual variability in the response to chronic stress remains incompletely explained in clinical physiology. Individuals exposed to comparable biological and environmental demands show radically different trajectories: some maintain physiological resilience, efficient recovery, and preserved cognitive function across decades; others accumulate progressive dysregulation that manifests first as a decline in adaptive capacity—often long before any diagnosable pathology appears—and ultimately culminates in chronic disease and accelerated biological aging. This divergence cannot be resolved by examining any single system in isolation.

Contemporary biomedical research has generated frameworks of considerable precision for specific physiological domains. In the domain of systemic regulation, allostasis theory formalized the concept of cumulative burden imposed by chronic stress on individual regulatory systems (McEwen and Stellar, 1993), while neurovisceral integration demonstrated that cardiac autonomic flexibility reflects central regulatory properties that transcend cardiovascular function (Thayer and Lane, 2009). In the domain of metabolism, mitochondrial stress physiology revealed that energy metabolism and psychological stress are molecularly intertwined—with mitochondria functioning not only as ATP producers but as critical mediators of stress signaling and biological adaptation (Picard and McEwen, 2018)—and the energy allocation resilience framework proposed that adaptive resilience is constrained by the finite allocation of metabolic resources across endocrine, immune, repair, and energetic demands under stress (Schuler et al., 2026). In the domain of biological plasticity, environmental epigenetics demonstrated that DNA methylation patterns can inscribe environmental history into the genome in persistent and functionally consequential ways (Meaney and Szyf, 2005), and second-generation epigenetic clocks—GrimAge and DunedinPACE—provide validated estimates of the rate of biological aging with independent predictive value for mortality and healthspan (Lu et al., 2019; Belsky et al., 2022). Beyond these domain-specific frameworks, physiological reserve theory has recognized that functional decline emerges when compensatory mechanisms across multiple organ systems are progressively exhausted—acknowledging reserve as an integrative property of the organism, although one conceptualized as the compensatory capacity remaining under accumulated damage rather than as a dynamic, prospectively measurable construct (Ferrucci and Fabbri, 2018). Each of these frameworks has made indispensable contributions to understanding biological adaptation—but each illuminates a single layer or a single dimension of reserve while leaving the relationship among layers underspecified (**Table 1**).

**Table 1:** Conceptual differentiation of the biological state hypothesis from related frameworks.

Concept	Central question	Unit of analysis	Temporal focus	Predictive target
Allostatic load	How much cumulative stress damage exists?	Individual systems	Cumulative	Disease risk
Neurovisceral integration	How flexible is autonomic regulation?	Autonomic nervous system (ANS)–brain axis	Momentary	Cardiovascular health
Epigenetic aging	How rapidly is biological aging occurring?	Methylome	Longitudinal	Mortality
Systems medicine	How do systems interact in disease?	Pathological networks	Variable	Disease mechanisms
Energy allocation resilience	How are finite metabolic resources allocated under stress to sustain resilience?	Metabolic–endocrine system	Dynamic	Resilience under metabolic demand
Physiological reserve	When do compensatory mechanisms across organ systems become exhausted?	Individual organ systems in cumulative decline	Longitudinal/cumulative	Frailty and functional disability
Biological state hypothesis	How much adaptive capacity remains available to the organism?	Integrated organism	Dynamic	Adaptive capacity trajectory

None of these frameworks, however, asks the question with which we began: given the organism’s current integrated physiological organization—not just the autonomic flexibility that neurovisceral integration isolates, not just the metabolic efficiency that mitochondrial stress physiology isolates, not just the epigenetic age that second-generation epigenetic clocks isolate, and not just the remaining organ reserve that physiological reserve theory isolates—how much adaptive capacity does it retain, and in which direction is that capacity trending? The evidence now available demonstrates that this question is not merely conceptual—the layers interact in bidirectional ways supported by existing evidence. In the direction of systemic regulation acting on the inner layers, the autonomic nervous system directly modulates hepatic glucose production, pancreatic insulin secretion, and adipose tissue lipolysis (establishing a well-documented SR→M pathway) (Wangler et al., 2026), and sustained hypothalamic-pituitary-adrenal (axis) (HPA) dysregulation and chronic sympathetic activation inscribe epigenetic traces in biological plasticity, methylating the promoters of *NR3C1* and brain-derived neurotrophic factor (*BDNF*) and accelerating telomere attrition (establishing an SR→BP pathway consistent with documented mechanisms) (Wang et al., 2026; Epel et al., 2004). In the direction of metabolism interacting bidirectionally, persistent metabolic dysregulation drives chronic low-grade inflammatory signaling (metaflammation) that suppresses autonomic flexibility, flattens the diurnal cortisol slope, and dysregulates circadian clock gene expression (establishing an M→SR pathway supported by existing evidence) (Hotamisligil, 2006), while the AMPK/SIRT1/PGC-1α axis links energetic state to epigenetic remodeling, DNA methylation, and *BDNF* expression (establishing an M→BP pathway consistent with documented mechanisms) (Chen et al., 2025; Godlewski and Dziaman, 2026). In the direction of biological plasticity feeding back upward, epigenetic modifications at *NR3C1* and *FKBP5* perpetuate HPA dysregulation and autonomic inflexibility (establishing a BP→SR pathway consistent with documented mechanisms) (Wang et al., 2026), while telomere–mitochondria crosstalk and the senescence-associated secretory phenotype progressively compromise bioenergetic capacity and metabolic flexibility (establishing a BP→M pathway supported by existing evidence) (Blackburn et al., 2015; Furman et al., 2019). These are six bidirectional interactions supported by existing evidence among regulatory, metabolic, and plasticity processes—operating simultaneously, continuously, and in mutual dependence.

The biological state hypothesis proposes that this integrated organization has a measurable emergent consequence: adaptive capacity. Biological state is defined as the dynamic expression of the functional order with which the organism engages its environment—continuously shaped by the interaction among systemic regulation, metabolism, and biological plasticity, and continuously challenged by environmental demands that tend, by nature, toward disorder. The central proposition is that adaptive capacity—the organism’s available margin to respond, recover, and adjust—is not the product of any single system. It emerges from the coherence of the integrated biological state. This distinguishes the biological state hypothesis (BSH) from frameworks that address systemic regulation specifically (allostatic load), which quantifies cumulative physiological cost retrospectively, and neurovisceral integration, which models autonomic flexibility as an isolated regulatory property; from frameworks that address metabolism (energy allocation resilience), which addresses resource distribution within the metabolic domain without integrating regulatory and plasticity dimensions; from frameworks that address biological plasticity (epigenetic clocks), which estimate the velocity of biological aging without capturing the organism’s remaining adaptive margin; and from physiological reserve theory, which frames reserve as a retrospective property rather than a prospective, dynamically measurable margin. The BSH asks a fundamentally different question: given the organism’s current integrated organization, how much adaptive reserve remains—and in which direction is that capacity evolving?

**Figure 1:**
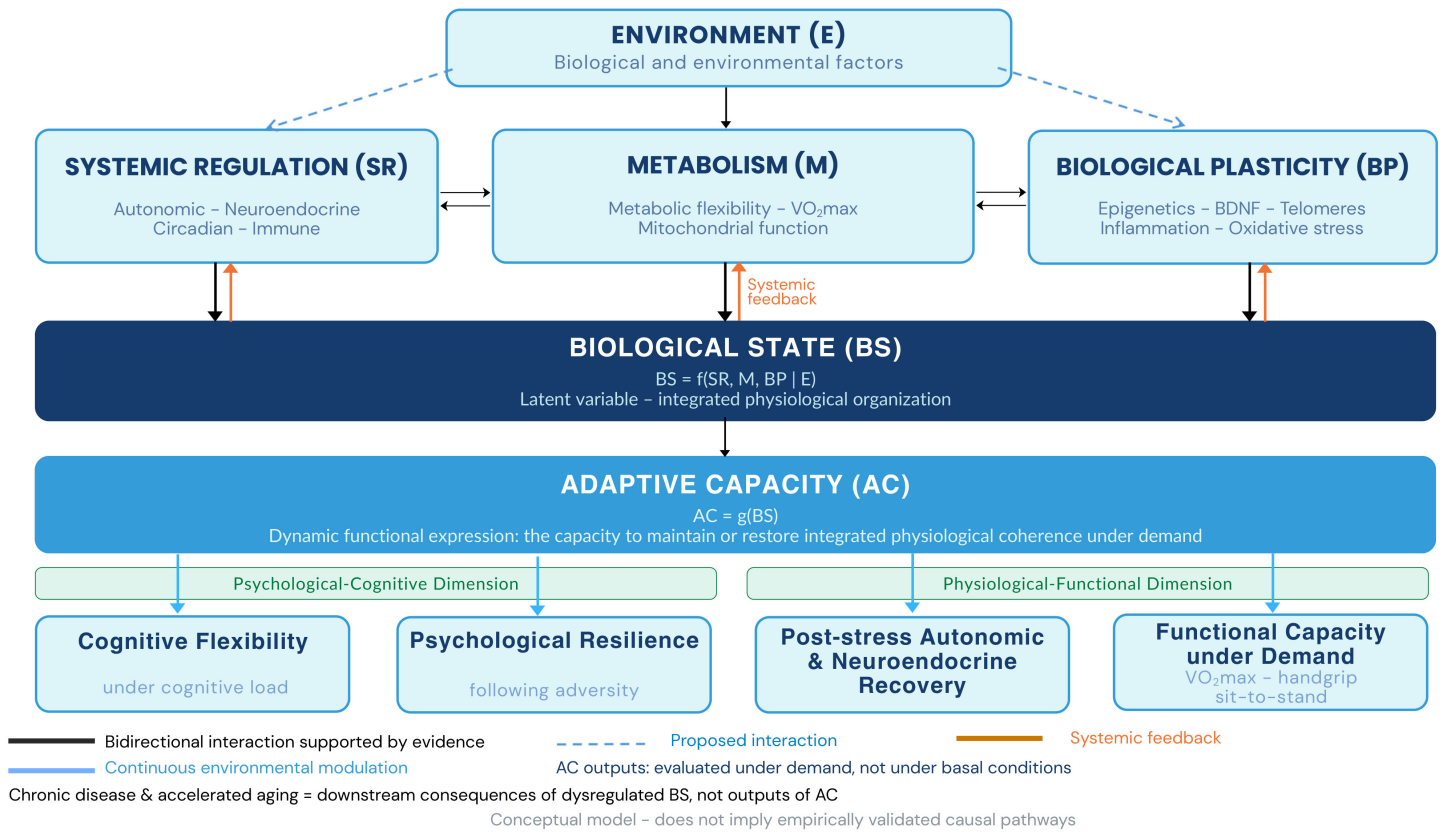
Conceptual representation of the biological state hypothesis. Biological state emerges from the dynamic, bidirectional, and non-linear interaction among three interdependent physiological layers—systemic regulation (SR), metabolism (M), and biological and epigenetic plasticity (BP)—continuously modulated by the environment (E). Systemic regulation integrates the autonomic, neuroendocrine, immune-inflammatory, and circadian axes. The environment acts predominantly through systemic regulation and metabolism, which together condition how biological plasticity is shaped over time. The resulting integrated physiological coherence acts as a systemic constraint on the organism’s adaptive capacity—an emergent functional property formally expressed as AC = g(BS). Chronic non-communicable disease and accelerated aging represent downstream consequences of a persistently dysregulated biological state, not the central object of the framework. Arrow types follow the figure legend: solid black arrows indicate bidirectional interactions supported by existing evidence, dashed arrows indicate proposed interactions, orange arrows indicate systemic feedback from biological state to its constituent layers, and light-blue arrows indicate continuous environmental modulation. The bidirectional coupling between adaptive capacity and biological state—whereby impaired adaptive capacity may itself further degrade biological state—is addressed in the text and must be examined in prospective longitudinal designs. This image represents a conceptual model intended to illustrate proposed organizational relationships and does not imply empirically validated causal pathways.

The central contribution of this framework is not the identification of new physiological domains. It is the proposal that the integrated organization of these domains constitutes a measurable systemic constraint on adaptive capacity (**Figure 1**)—one that can be operationalized, tested, and ultimately used to understand why some individuals maintain resilience across decades while others lose their adaptive margin long before disease becomes clinically apparent.

A distinction that recurs throughout this framework is stated here at the outset: biological state is assessed in basal, resting conditions, whereas adaptive capacity is evaluated in the organism’s response to an imposed demand—a difference that explains why some indicators later appear both as state markers and as functional outcomes.

## 2 Conceptual foundations of the biological state hypothesis

### 2.1 Biological state as an emergent physiological condition

Biological state is not a fixed physiological parameter—it is an emergent condition. It arises from the continuous, dynamic interaction among three interdependent layers: systemic regulation, encompassing the coordinated activity of the autonomic, neuroendocrine, immune-inflammatory, and circadian systems; metabolism, understood as the organism’s capacity to generate, allocate, and utilize energy in support of regulatory, repair, and adaptive processes; and biological plasticity, comprising both neuroplasticity and epigenetic–genomic plasticity, through which sustained environmental pressures are progressively inscribed into the organism’s biological architecture. None of these layers operates in isolation, and none can be fully understood without reference to the others.

This organization is not reducible to the individual’s genetic profile. The genotype defines potential vulnerabilities, but it is the dynamic interaction among the three layers—continuously modulated by the environment—that determines how, and to what degree, those vulnerabilities are phenotypically expressed. It has been argued that physiological function cannot be reduced to linear mechanisms operating within single organizational levels (Noble, 2012; Sterling, 2012). The biological state hypothesis extends this principle by proposing that the integrated organization of systemic regulation, metabolism, and biological plasticity constitutes a measurable systemic constraint on the organism’s adaptive capacity.

Operationally, biological state is proposed as a latent variable. Formally:

BS = f(SR, M, BP | E),

where BS represents biological state, SR represents systemic regulation, M represents metabolism, BP represents biological and epigenetic plasticity, and E represents environmental influences that continuously modulate all three layers. The function f() is not theoretically imposed but empirically determined. These equations are conceptual representations—not operational formulas—intended to express the proposed organizational relationships and guide future quantitative modeling.

Adaptive capacity may be represented as an emergent function of integrated biological state:

AC = g(BS).

When biological state maintains coherence among its three layers, adaptive capacity is broad, and the organism can respond, recover, and adjust effectively to the demands of its environment. When biological state deteriorates persistently, adaptive capacity progressively narrows—and it is this sustained narrowing that precedes and determines the emergence of chronic disease and accelerated biological aging, not the reverse.

As an initial operational framework, biological state may be modeled as a latent multidomain construct: BSI = w1(SR) + w2(M) + w3(BP). Initial implementations may assume equal weighting across domains. Future longitudinal studies may empirically derive these weights through latent variable models, structural equation modeling, Bayesian approaches, or other appropriate statistical methods. Multimodal machine learning approaches could subsequently be explored as complementary tools for modeling complex inter-domain interactions—not as the primary analytical framework, but as one among several appropriate methodological strategies depending on the specific research objective.

To avoid conceptual ambiguity, the hierarchy among the central constructs of the framework is stated explicitly and summarized in **Table 2**. Biological state constitutes the organism’s latent physiological organization—not directly observable, but approximable through multidomain indicators. Integrated physiological coherence describes the degree of synchronization among the three layers. Adaptive reserve represents the magnitude of adaptive capacity that coherence enables at any given moment. Adaptive capacity is the dynamic functional expression of that reserve—evaluated not in basal conditions but in the organism’s encounter with environmental demands, and manifested across two complementary dimensions, the psychological and cognitive dimension, reflected in cognitive flexibility, psychological resilience, and emotional regulation under load; and the physiological and functional dimension, reflected in post-stress autonomic recovery, neuroendocrine recovery, metabolic recovery, and sustained functional capacity under demand. Adaptive amplitude describes the range of possible responses available to the organism. In brief, adaptive reserve is the latent margin available to the organism, whereas adaptive capacity is the observed performance of that margin when the organism is actually challenged. These constructs are not synonymous—they represent distinct organizational levels of the same integrated system, from underlying physiological organization to observable functional expression. The concept of adaptive reserve as proposed here is conceptually distinct from physiological reserve as defined in geriatric and aging frameworks. Where physiological reserve quantifies the remaining compensatory capacity of individual organ systems under cumulative damage, adaptive reserve in this framework refers to the functional expression of integrated biological state coherence—a property that can expand or contract dynamically depending on the degree of organization among systemic regulation, metabolism, and biological plasticity, and that is not limited to older populations or disease contexts.

**Table 2:** Operational definitions of the central constructs of the biological state hypothesis.

Construct	Operational definition
Biological state (BS)	Dynamic physiological condition emerging from the continuous interaction among systemic regulation, metabolism, and biological plasticity, modulated by the environment. Operationalized as a latent variable. It functions as a systemic constraint on adaptive capacity—not determining it directly, but establishing the boundaries within which it can be expressed.
Integrated physiological coherence	State in which the three layers of biological state operate in a synchronized and mutually sustained manner. It does not equate to homeostasis nor to allostasis—it represents the dynamic relational organization among interdependent layers that enables the organism to maintain or restore physiological coherence when confronted with environmental demands, thereby preserving adaptive capacity and adaptive reserve.
Adaptive reserve	Magnitude of adaptive capacity that the organism’s integrated biological state enables at any given moment. It is not a fixed threshold but a dynamic gradient—broad when the three layers maintain coherence, and progressively diminishing when dysregulation persists across domains.
Adaptive capacity (AC)	Adaptive capacity is the dynamic functional expression of the organism’s ability to maintain or restore integrated physiological coherence when confronted with environmental demands. Evaluated not in basal conditions but in the organism’s encounter with the environment, across two complementary dimensions: psychological and cognitive, reflected in cognitive flexibility, psychological resilience, and emotional regulation under load; and physiological and functional, reflected in post-stress autonomic recovery, neuroendocrine recovery, metabolic recovery, and sustained functional capacity under demand. Its preservation is associated with health; its sustained reduction, with chronic disease and accelerated biological aging.
Adaptive amplitude	Range of physiological, cognitive, and behavioral responses available to the organism at any given moment, determined by the degree of integrated physiological coherence of its biological state. When coherence is high, adaptive amplitude is broad; when biological state deteriorates persistently, adaptive amplitude narrows progressively.
Systemic regulation (SR)	The organism’s capacity to maintain adaptive physiological coherence through the dynamic coordination of the autonomic, neuroendocrine, immune-inflammatory, and circadian systems.
Metabolism (M)	The organism’s capacity to generate, allocate, and utilize energy resources efficiently in support of regulatory, repair, and biological adaptation processes.
Biological plasticity (BP)	The organism’s capacity to remodel, maintain, and repair biological structures and functions through cellular, neural, epigenetic, and immunological mechanisms that enable sustained adaptation over time.

### 2.2 Systemic regulation

Systemic regulation is proposed as the organism’s capacity to maintain adaptive physiological coherence through the dynamic coordination of four interdependent axes: autonomic, neuroendocrine, immune-inflammatory, and circadian. These axes do not operate sequentially—they interact continuously and bidirectionally, such that dysregulation in any one propagates across the others and, as examined in Section 2.5, across the other layers of biological state.

In the autonomic domain, heart rate variability provides non-invasive measures of sympathetic–parasympathetic balance and its relationship with neuroendocrine regulation, cognitive function, and immune-inflammatory modulation (Thayer and Lane, 2009; Thayer et al., 2012).

In the neuroendocrine domain, the diurnal salivary cortisol profile—including the cortisol awakening response (CAR) and the diurnal decline slope—characterizes HPA axis activity and its regulatory capacity throughout the day (Clow et al., 2004; Pruessner et al., 1997). A flat diurnal slope has been consistently associated with greater allostatic load, immune-inflammatory dysregulation, and adverse health outcomes across multiple domains (McEwen, 2006; Adam et al., 2017).

In the immune-inflammatory domain, adequate regulation of inflammatory processes constitutes an essential component of physiological homeodynamics. Persistent alterations in the balance between pro-inflammatory and anti-inflammatory signals have been associated with systemic dysfunction, accelerated biological aging, and greater vulnerability to chronic non-communicable disease (Slavich and Irwin, 2014; Furman et al., 2019). Markers such as high-sensitivity C-reactive protein (hs-CRP), IL-6, TNF-α, and IL-10 provide complementary information about this regulatory dimension.

In the circadian domain, the synchronization of internal biological rhythms represents a fundamental component of systemic regulation. Persistent disruption of sleep–wake cycles and the temporal organization of physiological processes has been associated with metabolic, neuroendocrine, cardiovascular, and immunological alterations—establishing circadian integrity as a relevant dimension of the organism’s capacity to sustain coherent adaptive responses over time (Scheer et al., 2009; Czeisler and Gooley, 2007; Buxton and Marcelli, 2010).

### 2.3 Metabolism

Metabolism is proposed as the organism’s capacity to efficiently generate, allocate, and utilize metabolic energy in support of regulatory, repair, and adaptive processes. It constitutes the intermediate layer of biological state—receiving regulatory inputs from systemic regulation and conditioning, in turn, the processes of biological plasticity that depend on adequate energetic availability.

VO_2_max represents the most validated indicator of integrated mitochondrial oxidative capacity (Kodama et al., 2009; Brooks, 2018; Hawley et al., 2014). Mitochondria not only produce ATP but also participate in cellular stress signaling associated with biological adaptation—functioning as critical mediators between the regulatory demands imposed by systemic regulation and the plasticity processes that sustained adaptation requires (Picard and McEwen, 2018; Picard et al., 2016). Metabolic flexibility—assessed through fasting glucose, insulin, homeostatic model assessment of insulin resistance (HOMA-IR), glycated hemoglobin (hemoglobin A1c) (HbA1c) and the triglyceride:high-density lipoprotein (HDL) ratio—characterizes the organism’s capacity to alternate between energy substrates in response to physiological demand. The molecular pathways through which metabolic state actively conditions biological plasticity—and through which metabolic dysregulation propagates bidirectionally into systemic regulation—are examined in the context of the full inter-layer interaction model in Section 2.5.

### 2.4 Biological and epigenetic plasticity

Biological plasticity is proposed as the organism’s capacity to sustain physiological, cognitive, and regulatory adaptations in response to persistent demands—encompassing mechanisms of remodeling, maintenance, and biological repair that enable adaptation to be maintained over time.

Unlike systemic regulation, which reflects the dynamic coordination of ongoing physiological responses, biological plasticity relates to the more durable biological changes that such responses generate on the structure and function of the organism. It represents the sustained adaptation dimension of biological state—the layer through which the accumulated history of environmental demands, mediated through systemic regulation and metabolism, becomes inscribed in the organism’s biology and conditions its future adaptive responses.

This layer integrates two complementary dimensions. Neuroplasticity operates at the level of neural circuits, synaptic connectivity, and neurotrophic support across timescales of hours to weeks. Epigenetic–genomic plasticity operates at the level of gene expression regulation—through DNA methylation, histone modification, and non-coding RNA programs—across timescales of weeks to years, and in some cases across generations. These two dimensions are distinct in their mechanisms and temporal scales, but unified in their functional role: both are mechanisms through which sustained environmental pressures, mediated predominantly (although not exclusively) through systemic regulation and metabolism, are progressively inscribed into the organism’s biological architecture and condition its capacity for future adaptation. This shared functional principle justifies their integration within a single construct.

Second-generation epigenetic clocks such as GrimAge and DunedinPACE provide validated estimates of the rate of biological aging and have demonstrated growing predictive value for multiple health- and mortality-related outcomes (Lu et al., 2019; Belsky et al., 2022). Available evidence further suggests that prolonged exposure to chronic stress may be associated with accelerated telomere shortening (Blackburn et al., 2015; Epel et al., 2004). At the level of specific regulatory loci, sustained dysregulation of systemic regulation and metabolism has been associated with epigenetic modifications at genes including *NR3C1*, *FKBP5*, and *BDNF*—illustrating how the upper layers of biological state leave molecular traces in biological plasticity that alter the organism’s future regulatory sensitivity (Wang et al., 2026).

Serum *BDNF* represents the most studied peripheral indicator of neuroplasticity, linking exercise, metabolic state, and cognitive function through epigenetic mechanisms at the *BDNF* promoter (Bathina and Das, 2015; Castrén and Hen, 2013). Inflammatory markers—including IL-6, hs-CRP, TNF-α, and IL-10—appear in this framework in two distinct functional roles. In Section 2.2, they serve as indicators of immune-inflammatory regulation within systemic regulation. Here, in the context of biological plasticity, they are considered indicators of the organism’s capacity for sustained biological repair, remodeling, and tissue maintenance—processes that determine whether the organism can preserve its adaptive architecture over time. These are complementary, not contradictory, readings of the same markers: one reflecting acute regulatory function and the other reflecting the cumulative biological cost of sustained inflammatory dysregulation on long-term plasticity (Furman et al., 2019).

### 2.5 Dynamic interaction among domains and the pathway to adaptive reserve depletion

The three layers of biological state do not operate in sequence—they engage in continuous, bidirectional, and non-linear interactions that collectively determine the organism’s integrated physiological coherence and its available adaptive reserve. The preceding sections described each layer and its principal indicators. This section examines how those layers interact over time—and how their progressive dysregulation narrows adaptive reserve and opens the pathway toward chronic non-communicable disease and accelerated biological aging.

When the organism encounters biological or environmental demands, systemic regulation activates coordinated autonomic, neuroendocrine, immune-inflammatory, and circadian responses. These responses temporarily increase energetic demand on metabolic systems—the autonomic nervous system directly modulates hepatic glucose production, pancreatic insulin secretion, and adipose tissue lipolysis, establishing the SR→M pathway (Wangler et al., 2026). When recovery is adequate, integrated physiological coherence is restored, adaptive reserve is preserved, and biological plasticity may even be strengthened through hormetic mechanisms—including mitochondrial biogenesis activated through the Nrf2/PGC-1α/PINK1-Parkin pathway in response to moderate physiological challenges (Da et al., 2024). This is the normal adaptive cycle.

When demands persist and recovery is insufficient, a different trajectory unfolds. Sustained HPA dysregulation and chronic sympathetic activation inscribe epigenetic traces in biological plasticity—methylating the promoters of *NR3C1* and brain-derived neurotrophic factor (*BDNF*) and accelerating telomere attrition—establishing the SR→BP pathway (Wang et al., 2026; Epel et al., 2004). Simultaneously, metabolic dysregulation driven by sustained energetic demand activates pro-inflammatory pathways including NF-κB signaling and NLRP3 inflammasome activation—generating metaflammation that suppresses vagal activity, flattens the diurnal cortisol slope, and dysregulates circadian clock gene expression—establishing the M→SR pathway (Hotamisligil, 2006). The AMPK/SIRT1/PGC-1α axis links the deteriorating energetic state to epigenetic remodeling, altering DNA methylation, histone acetylation, and *BDNF* expression—establishing the M→BP pathway (Chen et al., 2025; Godlewski and Dziaman, 2026). Over time, biological plasticity feeds back into both systemic regulation and metabolism: epigenetic modifications at *NR3C1* and FKBP5 perpetuate HPA dysregulation and autonomic inflexibility (establishing the BP→SR pathway) (Wang et al., 2026), while telomere–mitochondria crosstalk and the senescence-associated secretory phenotype progressively compromise bioenergetic capacity and metabolic flexibility (establishing the BP→M pathway) (Blackburn et al., 2015; Furman et al., 2019).

Within this framework, allostatic load may be interpreted as one of the mechanisms by which repeated perturbations accumulate physiological cost across the layers of biological state (McEwen and Stellar, 1993; McEwen, 1998). Chronic non-communicable disease and accelerated biological aging represent the downstream clinical expression of this sustained loss of integrated biological state—not isolated events with independent origins, but the convergent consequences of a system whose coherence has been progressively eroded.

The proposed interactions should be interpreted not as demonstrated causal hierarchies but as a hypothesized dynamic coupling among interdependent physiological domains—one whose temporal direction and bidirectional dynamics are examined in detail in Sections 4.2 and 5.1.

### 2.6 Integrated physiological coherence

Integrated physiological coherence constitutes the central mechanism through which biological state acts as a systemic constraint on the organism’s adaptive capacity. It does not equate to homeostasis (which refers to the maintenance of stability in individual physiological variables) nor to allostasis (which describes the capacity of a system to predictively adjust in response to stress) (Sterling, 2012). Integrated physiological coherence describes the relational organization among the three layers of biological state that makes sustained adaptive adjustment possible across time, demand, and biological domains.

Integrated physiological coherence is not a binary state but a continuous gradient that conditions the organism’s adaptive reserve and, through it, its adaptive capacity. When coherence among systemic regulation, metabolism, and biological plasticity is high, adaptive reserve is broad, the organism can absorb and respond to a wide range of demands, and health is preserved. When coherence is persistently reduced, adaptive reserve progressively diminishes, the organism becomes increasingly vulnerable to demands that it would previously have absorbed without consequence, and the conditions are established for the emergence of chronic non-communicable disease and accelerated biological aging. This is the central clinical implication of the biological state hypothesis: health and disease are not binary states separated by a diagnostic threshold—they represent positions along a continuum of integrated physiological coherence that can, in principle, be estimated, monitored, and intervened upon.

## 3 Integrated biomarker panel for the operationalization of biological state

Biological state is proposed as a latent physiological construct that emerges from the dynamic interaction among systemic regulation, metabolism, and biological plasticity. Given its multidomain nature, no individual biomarker can adequately capture the complexity of the construct. Accordingly, a tiered operationalization strategy is proposed, organized into two levels: a minimum operational core and a clinical expansion layer. This structure reflects the progressive nature of construct validation and responds to the practical reality that different research and clinical contexts will require different levels of physiological resolution.

The biomarkers presented in this section should be understood as an initial operational proposal intended to facilitate future empirical validation phases. They do not constitute a definitive or exhaustive list, and their selection may be refined as available evidence evolves. The principal contribution of this work is the theoretical framework; the panel illustrates a possible strategy for operationalizing the construct. It is explicitly stated that the biological state index, the proposed biomarker panel, and the clinical translation pathway described in this manuscript remain hypothetical constructs until prospectively validated in independent cohorts.

### 3.1 Minimum operational core

The minimum operational core proposes a reduced set of biomarkers, representative of the three layers of biological state, that can be implemented in clinical and research contexts with standard diagnostic resources (**Table 3**). Each marker was selected on the basis of three criteria: documented validity as an indicator of its respective domain, independent predictive value for health-related outcomes, and practical feasibility in diverse healthcare settings. Following empirical validation, the minimum core is intended to remain parsimonious: a marker that fails to add predictive value beyond lower-cost indicators of the same layer—for example, if serum *BDNF* or the epigenetic clocks do not improve prediction over cheaper markers—would be reassigned to the clinical expansion layer rather than retained in the core.

**Table 3:** Integrated biomarker panel—minimum operational core and clinical expansion layer.

Layer	Subdomain	Minimum operational core	Clinical expansion layer
**Systemic regulation**	Autonomic	HRV (wearable device)	Advanced HRV metrics (RMSSD, SDNN, and LF/HF), post-exertion heart rate recovery
	Neuroendocrine	Diurnal salivary cortisol profile (CAR + slope)	4-point salivary cortisol, DHEA-S, cortisol:DHEA-S ratio
	Immune-inflammatory	hs-CRP	NLR, IL-6, TNF-α, IL-10
	Circadian	Sleep patterns and circadian regularity (actigraphy or validated instruments)	Melatonin, circadian regularity indices, sleep–wake variability
**Metabolism**	Oxidative capacity	VO_2_max	—
	Metabolic flexibility	HOMA-IR, TG/HDL ratio	Fasting glucose, insulin, HbA1c, complete lipid panel
	One-carbon metabolism	—	Homocysteine, holotranscobalamin (active B12), folate, MMA
	Nutritional/energy status	—	Ferritin, vitamin D
	Systemic metabolic function	—	Hepatic function (GGT, AST, and ALT); creatinine and estimated GFR
	Advanced bioenergetics	—	Coenzyme Q10; lactate:pyruvate ratio
**Biological plasticity**	Biological aging	GrimAge or DunedinPACE (epigenetic clocks)	Leukocyte telomere length, telomerase activity; Horvath (2013) and Hannum et al. (2013) clocks
	Neuroplasticity	Serum *BDNF*	IGF-1
	Molecular integrity	—	Urinary 8-OHdG
	Targeted epigenetics	—	Methylation at candidate loci (*NR3C1*, *FKBP5*, *BDNF*, and *SLC6A4*); high-resolution methylation sequencing

#### 3.1.1 Systemic regulation—core markers

Heart rate variability (HRV) constitutes the most validated non-invasive indicator of autonomic regulation available in the clinical context. It reflects the dynamic balance between sympathetic and parasympathetic activities, and its relationship with neuroendocrine function, immune-inflammatory modulation, and cognitive performance has been documented across multiple domains (Thayer and Lane, 2009; Thayer et al., 2012). HRV can be obtained through wearable devices, making it accessible and longitudinally trackable.

The diurnal salivary cortisol profile, including the CAR and the diurnal decline slope, characterizes HPA axis activity and its regulatory capacity throughout the day. A flat diurnal slope has been consistently associated with greater allostatic load, immune-inflammatory dysregulation, and adverse health trajectories (McEwen, 2006; Adam et al., 2017; Clow et al., 2004).

hs-CRP provides a validated and widely accessible indicator of systemic immune-inflammatory regulation. Persistent alterations in inflammatory balance have been associated with systemic dysfunction, accelerated biological aging, and greater vulnerability to chronic non-communicable disease (Slavich and Irwin, 2014; Furman et al., 2019).

Sleep patterns and circadian regularity, assessed through validated instruments or actigraphy, capture the temporal organization of the biological system. Persistent circadian disruption has been associated with metabolic, neuroendocrine, cardiovascular, and immunological alterations—establishing circadian integrity as a functionally relevant dimension of systemic regulation (Scheer et al., 2009; Czeisler and Gooley, 2007).

#### 3.1.2 Metabolism—core markers

VO_2_max represents the most validated integrated indicator of mitochondrial oxidative capacity and bioenergetic performance. Its independent predictive value for all-cause mortality, cognitive function, and biological aging trajectory is among the strongest in the clinical literature (Kodama et al., 2009; Brooks, 2018; Hawley et al., 2014).

HOMA-IR provides a clinically accessible estimate of insulin sensitivity—the central axis of metabolic flexibility and a key determinant of the organism’s capacity to allocate energetic resources efficiently under demand.

The triglyceride:HDL ratio (TG/HDL) constitutes an accessible indicator of metabolic flexibility and global cardiometabolic health, capturing the organism’s capacity to manage lipid substrates in response to physiological demand.

#### 3.1.3 Biological plasticity—core markers

Second-generation epigenetic clocks (GrimAge or DunedinPACE) provide validated estimates of biological aging trajectory and pace—reflecting the integrated accumulation of environmental and physiological influences on the organism’s epigenetic architecture. Their predictive value for mortality, healthspan, and disease risk is the strongest among available aging biomarkers (Lu et al., 2019; Belsky et al., 2022).

Serum *BDNF* constitutes the most studied peripheral indicator of neuroplasticity, linking physical activity, metabolic state, and cognitive adaptability through epigenetic mechanisms at the *BDNF* promoter (Bathina and Das, 2015; Castrén and Hen, 2013).

### 3.2 Clinical expansion layer

The clinical expansion layer includes biomarkers that broaden the physiological resolution of each domain and may be incorporated in clinical contexts with greater diagnostic resources or in research protocols requiring more detailed domain characterization. Within this layer, markers are distinguished between those with established clinical utility and those with primarily research or exploratory value.

#### 3.2.1 Systemic regulation—expansion

Autonomic domain: Advanced HRV metrics [root mean square of successive differences (heart rate variability) (RMSSD), standard deviation of NN (normal-to-normal) intervals (heart rate variability) (SDDN), and low frequency to high frequency ratio (heart rate variability spectral analysis) (LF/HF) ratio] and post-exertion heart rate recovery as an indicator of autonomic resilience under physiological demand.

Neuroendocrine domain: Four-point daily salivary cortisol, dehydroepiandrosterone sulfate, and cortisol: dehydroepiandrosterone sulfate (DHEA-S) ratio—reflecting the balance between catabolic and anabolic neuroendocrine regulation and its relationship with adaptive reserve.

Immune-inflammatory domain: Neutrophil-to-lymphocyte ratio (NLR), IL-6, TNF-α, and IL-10—providing information about the directionality and balance of the inflammatory response beyond systemic burden captured by hs-CRP alone.

Circadian domain: Actigraphy-derived sleep–wake variability indices, melatonin, and validated circadian regularity indices.

#### 3.2.2 Metabolism—expansion

Glucose metabolism: Fasting glucose, insulin, and HbA1c.

One-carbon metabolism: Homocysteine, holotranscobalamin (active B12), folate, and methylmalonic acid (MMA). This subdomain is included because one-carbon metabolism regulates the provision of methyl groups—through the folate and methionine cycles—for DNA methylation and histone modification reactions fundamental to biological plasticity (Friso et al., 2017; Dang et al., 2024). Elevated homocysteine, by altering the S-adenosylmethionine to S-adenosylhomocysteine (SAM: SAH) ratio, directly impairs methylation capacity and epigenetic regulation—establishing one-carbon metabolism as a critical metabolic bridge between the metabolism layer and biological plasticity (Joshi and Jadavji, 2024).

Nutritional and energy status: Ferritin and vitamin D—included as indicators of micronutrient availability that condition mitochondrial function, immune regulation, and epigenetic processes.

Systemic metabolic function: Complete lipid panel, hepatic function [gamma-glutamyl transferase (GGT), aspartate aminotransferase (AST), and alanine aminotransferase (ALT)], and creatinine and estimated glomerular filtration rate (GFR)—reflecting the functional integrity of metabolically critical organ systems as indicators of the organism’s overall energetic and metabolic competence.

Advanced bioenergetics: Coenzyme Q10 and lactate:pyruvate ratio—providing direct indicators of mitochondrial function and redox status appropriate for specialized research contexts.

#### 3.2.3 Biological plasticity—expansion

Biological aging: Leukocyte telomere length and telomerase activity (Blackburn et al., 2015; Epel et al., 2004). Reference epigenetic clocks—Horvath (2013) and Hannum et al. (2013)—are retained as methodological reference points for comparison with second-generation clocks in validation studies.

Molecular integrity: Urinary 8-OHdG—an indicator of oxidative DNA damage reflecting the balance between biological wear and repair capacity.

Biological repair and maintenance: IGF-1—reflecting anabolic and reparative processes associated with sustained biological adaptation.

Targeted epigenetics: Methylation at candidate regulatory loci (*NR3C1*, *FKBP5*, *BDNF*, and *SLC6A4*); high-resolution methylation analysis through next-generation sequencing reserved for validation studies with appropriate infrastructure.

### 3.3 Considerations for standardization and construction of the biological state index

The dynamic nature of biological state implies that many of the proposed biomarkers exhibit intraindividual variability dependent on measurement timing, physiological context, and environmental conditions—including fasting status, sleep quality in the preceding night, recent physical activity, acute illness, medication use, and menstrual cycle phase. Future validation studies will need to establish rigorous standardization protocols to minimize unwanted variability and improve comparability across cohorts and longitudinal assessments.

The future construction of the biological state index (BSI) will require formal validation procedures, including correlation analysis among proposed indicators, dimensionality reduction, latent variable modeling, and longitudinal evaluation of predictive performance. Initial implementations may employ structural equation modeling or Bayesian approaches to estimate domain weights empirically. Multimodal machine learning may be explored as a complementary analytical tool for modeling complex inter-domain interactions once adequate sample sizes and external validation cohorts are available—not as the primary modeling framework, but as one among several methodologically appropriate strategies.

The proposed panel should be understood as an operational starting point for theory validation—not as a definitive formulation of the construct. It is explicitly acknowledged that the biological state index remains a hypothetical construct until prospectively validated in independent populations across diverse clinical and demographic contexts.

## 4 Physiological and translational implications

### 4.1 Biological state and adaptive capacity

Adaptive capacity may be understood as the functional expression of the organism’s ability to respond, recover, and maintain its functioning in the face of the continuous demands of the environment. From the perspective of the biological state hypothesis, this capacity does not depend on any single physiological system—it emerges from the integrated organization of the regulatory, metabolic, and biological plasticity processes that constitute the individual’s biological state. When that organization is preserved, adaptive capacity is broad, and the organism can absorb, respond to, and recover from a wide range of environmental demands. When biological state deteriorates persistently, adaptive capacity progressively narrows—and it is this narrowing that opens the pathway toward chronic non-communicable disease and accelerated biological aging, not the reverse.

It is essential to distinguish biological state from adaptive capacity—they are related but conceptually distinct constructs. Biological state is the integrated physiological organization of the organism, assessed in basal conditions through the indicators of the minimum operational core described in Section 3: HRV, diurnal cortisol profile, hs-CRP, sleep regularity, VO_2_max, HOMA-IR, TG/HDL ratio, epigenetic clocks, and serum *BDNF*. These indicators capture the state of the system at rest—how organized, coherent, and functionally available each layer is before any challenge is imposed. Adaptive capacity, by contrast, is not assessed in basal conditions. It is evaluated in the organism’s functional response when a demand is actually imposed—in the encounter with the environment.

Adaptive capacity manifests across two complementary dimensions. The first is the psychological and cognitive dimension: how the mind and brain respond to environmental demands—reflected in cognitive flexibility under load, psychological resilience following adversity, and emotional regulation capacity under sustained stress, evaluated through validated neuropsychological instruments and resilience scales. The second is the physiological and functional dimension: how the body responds and recovers when challenged—reflected in the speed and completeness of autonomic recovery after a standardized stressor, assessed through post-stress HRV recovery curves; in neuroendocrine recovery, assessed through the cortisol return slope following HPA activation; in metabolic recovery, assessed through lactate clearance following standardized physical challenge; and in sustained functional capacity under demand, approximated through VO_2_max in an effort protocol, handgrip dynamometry, or sit-to-stand performance (Leong et al., 2015; Kodama et al., 2009). The trajectory of biological aging—estimated longitudinally through DunedinPACE—provides an additional expression of adaptive capacity over time: whether the organism’s biological reserve is expanding, stable, or contracting in response to its accumulated life demands.

It is important to note that some instruments—such as HRV or VO_2_max—appear both as indicators of biological state in Section 3 and as expressions of adaptive capacity here. This is not a contradiction. When HRV is measured in basal, resting conditions, it reflects the current state of autonomic regulation—an indicator of the SR layer of biological state. When HRV is measured as the recovery curve following a standardized physiological challenge, it reflects how the organism’s adaptive capacity is expressed under demand. The same instrument, in different measurement contexts, captures different levels of the same system.

Adaptive reserve may be interpreted as the magnitude of adaptive capacity that the organism’s integrated physiological organization enables at any given moment. It is not a fixed threshold but a dynamic gradient—conditioned by the degree of coherence among the three layers of biological state and continuously modulated by the demands of the environment. An organism with high biological state coherence has a broad adaptive reserve and can face a wide range of demands while preserving functional integrity. An organism with a persistently deteriorated biological state has a narrow adaptive reserve—demands that others absorb without consequence may exceed its available margin, leading to disproportionate physiological cost and, progressively, to the clinical expression of chronic disease and accelerated biological aging.

### 4.2 Biological state, physiological dysregulation, and the pathway to chronic disease and accelerated aging

The biological state hypothesis proposes a temporal physiological sequence through which repeated biological and environmental exposures progressively shape the organism’s integrated state and, through it, its adaptive capacity. The bidirectional inter-layer interactions that underpin this sequence—SR⇔M, SR⇔BP, and M⇔BP—and their molecular mechanisms were detailed in Section 2.5. Rather than restating them, this section examines what that architecture implies clinically: how progressive inter-layer dysregulation narrows adaptive reserve, why the resulting trajectory is neither fixed nor unidirectional, and where it can be interrupted.

Under moderate demand followed by adequate recovery, the organism completes the normal adaptive cycle: integrated physiological coherence is restored, adaptive reserve is preserved, and biological plasticity may even be strengthened through hormesis. This is the physiological basis of adaptation to exercise, dietary variation, and controlled environmental challenge—the constructive expression of the same inter-layer coupling that, under sustained strain, becomes maladaptive.

When demands persist, and recovery is repeatedly insufficient, that coupling turns self-reinforcing—metabolic compromise feeding back into systemic regulation through metaflammation and epigenetic remodeling of stress-response loci—so that coherence erodes and adaptive reserve progressively narrows. Critically for clinical translation, this trajectory is not linear. Because the three layers are coupled, it can be slowed, arrested, or reversed by interventions targeting any one of them; this reversibility, rather than the accumulation of fixed damage, is what makes biological state a clinically actionable target.

Left unaddressed, however, the trajectory favors the progressive depletion of adaptive reserve, whose downstream clinical expression is chronic non-communicable disease and accelerated biological aging—convergent consequences of a system whose coherence has been progressively eroded, rather than isolated events with independent origins. Allostatic load, in this framing, is one mechanism by which repeated perturbations accumulate physiological cost across the layers of biological state (McEwen and Stellar, 1993).

Evidence from chronic-stress contexts is consistent with this framework. Sustained neuroendocrine dysregulation—reflected in alterations of the diurnal cortisol slope, the cortisol awakening response, and the cortisol:DHEA-S ratio—has been associated with metabolic, inflammatory, circadian, and epigenetic changes (McEwen, 2006; Picard and McEwen, 2018; Adam et al., 2017). Research on the biological consequences of chronic military stress has further described associations among persistent neuroendocrine alterations, sustained immune-inflammatory activation, and changes in adaptation-related mechanisms—including pathways linked to *NR3C1, FKBP5, BDNF*, and IL-6—illustrating how sustained dysregulation across the layers of biological state leaves molecular traces in biological plasticity that condition future adaptive responses (Laván et al., 2026).

These interactions are framed not as demonstrated causal hierarchies but as a hypothesized dynamic coupling among interdependent physiological domains. Their temporal direction—and in particular the bidirectional relationship between biological state and adaptive capacity, in which diminished adaptive capacity may itself further erode biological state—must be established through prospective longitudinal designs with repeated multidomain measurements.

### 4.3 Analytical framework for the assessment of biological state

Given the multidimensional, dynamic, and potentially non-linear nature of biological state, its assessment requires analytical strategies capable of integrating information from multiple physiological domains simultaneously. No single statistical or computational approach is sufficient for all stages of development and validation of the construct—the appropriate method depends on the specific research objective.

During the initial phases of construct characterization, dimensionality reduction approaches, latent profile analysis, and structural equation models are well suited for exploring the underlying organization of the proposed domains and evaluating the plausibility of a common latent variable. For the study of relationships among domains and their temporal evolution, mixed-effects longitudinal models and Bayesian approaches offer important advantages for capturing individual trajectories, uncertainty, and biological heterogeneity.

Once the structure and validity of the construct have been established, multimodal machine learning approaches may be employed as complementary tools for modeling complex inter-domain interactions and evaluating predictive capacity in independent cohorts. Their use must be accompanied by external validation, calibration assessment, explicit handling of missing data, and systematic comparison against simpler and more interpretable baseline models. Prediction 4 of this framework specifically requires that integrated multidomain models outperform single-domain models in independent validation cohorts—not only in internal cross-validation.

For future clinical applications, the practical utility of the model will depend not only on predictive performance but also on the interpretability, reproducibility, and feasibility of implementation across diverse healthcare contexts.

### 4.4 Translational and clinical perspectives

The biological state hypothesis offers a conceptual foundation for a shift in how physiological assessment is approached in clinical and research contexts—from the evaluation of individual systems in isolation toward the characterization of the organism’s integrated physiological organization and its available adaptive reserve.

Potential applications include the development of integrated biological state indices capable of estimating the organism’s current adaptive reserve, the identification of physiological profiles associated with different adaptive capacity trajectories before overt pathology appears, the design of multidomain interventions oriented toward restoring or preserving integrated physiological coherence across the three layers simultaneously, and longitudinal monitoring of biological state as a guide for clinical decision-making in prevention and chronic disease management. The framework also has implications for understanding why individuals with comparable clinical profiles respond differently to the same intervention—a question that future stratified intervention studies could directly test.

These applications must be considered prospective. The biological state index, the proposed biomarker panel, and the clinical translation pathway described in this manuscript remain hypothetical constructs until prospectively validated in independent populations across diverse clinical and demographic contexts. The clinical utility of the framework depends on future validation studies that establish the stability, reproducibility, predictive value, and interpretability of biological state measures.

#### 4.4.1 Proposed pathway for validation and clinical translation

Phase 1: Physiological construct validation. Empirically demonstrate that the integration of systemic regulation, metabolism, and biological plasticity can be represented as a multidomain, coherent, and reproducible physiological construct approximable through integrated biomarker panels.
Phase 2: Development and validation of the BSI. Construct an integrated index capable of reproducibly estimating the organism’s biological state and evaluate its stability, reliability, and validity in independent cohorts.
Phase 3: Physiological stratification and predictive capacity. Determine whether the BSI enables the identification of differentiated adaptive capacity trajectories, providing additional predictive information relative to individual biomarkers and conventional physiological models.
Phase 4: Clinical implementation and longitudinal monitoring. Evaluate the utility of the BSI for longitudinal monitoring of biological state and adaptive capacity, and for guiding multidomain interventions aimed at preserving or restoring integrated physiological coherence, once clinical validity has been demonstrated.

The realization of this pathway depends, however, on addressing a set of conceptual, methodological, and translational limitations that the current theoretical proposal has not yet resolved—and that define the critical challenges for the next phase of the development of the biological state hypothesis.

## 5 Limitations and future directions

### 5.1 Conceptual limitations

The biological state hypothesis constitutes a theoretical, integrative, and falsifiable proposal. The interactions posited among systemic regulation, metabolism, and biological plasticity have not yet been empirically validated as an integrated physiological system. Consequently, the present work does not establish causal relationships—it proposes a conceptual framework whose validity must be examined through longitudinal, multidomain, and prospective studies. This is not a weakness of the proposal but a defining characteristic of theoretical frameworks in systems physiology: they generate the research questions that empirical investigation must answer.

The operationalization of biological state through multidomain indicators will require conceptual, psychometric, and physiological validation in independent cohorts. The biomarker selection presented in this work should be considered illustrative and subject to refinement as available evidence evolves. The optimal composition of each physiological domain will need to be established empirically, and redundancy among indicators will need to be formally assessed through dimensionality reduction and latent variable modeling procedures.

Adaptive capacity, as conceptualized in this framework, may share elements with previously described constructs—including different forms of physiological and psychological resilience, functional capacity, and allostatic reserve. However, the present hypothesis proposes a specific conceptual distinction: adaptive capacity is not equivalent to resilience as a trait, nor to functional capacity as a static measure, nor to allostatic reserve as a retrospective index of physiological wear. Rather, it is defined as the dynamic functional expression of integrated biological state—evaluated in the organism’s encounter with environmental demands, not in basal conditions. The term adaptive capacity has been used in other scientific traditions—including social–ecological systems research and geriatric medicine—with related but distinct meanings. In the present framework, it refers specifically to the organism’s capacity to respond, recover, and reorganize its physiological coherence when confronted with a demand. Future studies will need to formally establish its discriminant validity relative to existing resilience and functional constructs, its convergent validity with established measures, and its incremental predictive contribution. The relationship between adaptive capacity as defined here and physiological reserve as conceptualized in aging frameworks will also require explicit discriminant validation, given the conceptual proximity of both constructs in their reference to the organism’s available functional margin.

The temporal direction of the relationship between biological state and adaptive capacity remains uncertain and constitutes one of the central open questions of this framework. It is plausible that deterioration of biological state conditions subsequent reductions in adaptive capacity—the primary direction proposed by the hypothesis. However, it is equally plausible that reduced adaptive capacity, through inactivity, poor sleep, stress amplification, and reduced recovery, progressively worsens biological state. These bidirectional dynamics are not a contradiction of the framework—they are a prediction of it. Resolving them will require prospective longitudinal designs with repeated multidomain measurements capable of modeling the temporal dynamics between both constructs with sufficient resolution.

### 5.2 Methodological limitations

The multidomain characterization of biological state poses methodological and operational challenges related to infrastructure, costs, and logistical complexity. The implementation of the framework will require standardized protocols for the acquisition, processing, and integration of biomarkers obtained through different diagnostic platforms.

As noted in Section 3.3, the dynamic nature of biological state implies that many proposed biomarkers exhibit intraindividual variability dependent on measurement timing and physiological context. From a methodological standpoint, this variability has direct implications for study design—requiring standardized assessment windows, controlled pre-measurement conditions, and explicit documentation of contextual factors, such as fasting status, prior sleep quality, recent physical activity, acute illness, medication use, and menstrual cycle phase, in order to ensure comparability across participants and across repeated longitudinal assessments.

In longitudinal studies based on real clinical data, biomarker absence is not necessarily random. The potentially informative nature of missing data must be explicitly considered during the design of analytical models and validation processes—avoiding the assumption of completely random absence mechanisms when this condition cannot be demonstrated. Appropriate missing data strategies, including multiple imputation and mixed-effects models that accommodate irregular measurement schedules, will be necessary.

The availability and standardization of some advanced biomarkers may vary considerably among institutions and healthcare systems. Future implementations of the framework will need to balance physiological depth, clinical feasibility, reproducibility, and accessibility, preserving the model’s conceptual coherence without compromising its applicability across diverse healthcare contexts.

### 5.3 Translational limitations

The translational applicability of the biological state hypothesis will depend on the identification and validation of operational biomarker cores capable of adequately representing the three domains while maintaining a balance between physiological depth, clinical feasibility, and implementation cost. The minimum operational core proposed in Section 3 represents an initial attempt to achieve this balance—but its clinical utility remains to be demonstrated in prospective studies.

The clinical utility of the framework will further depend on measures derived from biological state demonstrating reproducibility, temporal stability, and incremental predictive capacity relative to conventional physiological assessment strategies. A biological state index that does not add predictive value beyond existing clinical tools would not justify the additional complexity of its implementation.

The generalization of the model will require external validation studies in independent cohorts, populations with different biological and clinical characteristics, and different healthcare systems. It is explicitly stated that the biological state index, the proposed biomarker panel, and the clinical translation pathway described in this manuscript remain hypothetical constructs until prospectively validated in independent populations.

### 5.4 Future directions

The limitations identified above delineate a progressive research program for the conceptual, physiological, and clinical validation of the biological state hypothesis. In a first stage, studies should evaluate whether the integration of systemic regulation, metabolism, and biological plasticity can be represented through a reproducible and coherent latent construct using multidomain biomarker panels, employing structural equation modeling and latent variable approaches to determine whether the proposed domains share sufficient common variance to justify their integration.

A second stage should longitudinally examine the stability of the construct, its relationship with adaptive reserve, and its capacity to explain adaptive capacity trajectories in different populations—establishing whether changes in biological state precede observable modifications in adaptive capacity, which is the central temporal prediction of the framework.

Subsequently, the predictive performance of integrated biological state must be compared against individual biomarkers and existing physiological models—including allostatic load indices, epigenetic aging measures, neurovisceral integration models, and physiological reserve frameworks—using structural equation models, longitudinal mixed-effects models, Bayesian approaches, and multimodal machine learning as complementary strategies according to the specific objective of each study.

Finally, multicenter external validation studies will need to establish the reproducibility, generalizability, clinical utility, and incremental predictive value of the proposed framework in diverse populations and healthcare contexts, including populations from low- and middle-income settings where the burden of chronic non-communicable disease is the highest.

A particularly promising direction for future investigation concerns the development of dynamic assessments of adaptive capacity through standardized physiological challenge protocols. The present framework operationalizes biological state through indicators assessed in basal, resting conditions—capturing the organism’s integrated physiological organization at a given moment. A complementary future construct would assess adaptive capacity not through basal indicators but through the organism’s dynamic response to a controlled challenge—characterizing the velocity, completeness, and quality of physiological recovery across autonomic, neuroendocrine, metabolic, and cognitive domains following a standardized stressor. The central empirical question such an approach would address is whether biological state, assessed in basal conditions, predicts the form and quality of the organism’s recovery curve when confronted with a standardized demand—thereby establishing a prospective link between the organism’s integrated physiological organization and its functional adaptive behavior under real environmental pressure. This direction represents one of the most scientifically promising extensions of the present framework and will be the focus of subsequent research programs.

The four-phase validation and clinical translation pathway described in Section 4 provides the operational roadmap for addressing these limitations progressively—and the five falsifiable predictions presented in Section 6 define the specific empirical tests through which the validity of the biological state hypothesis will ultimately be determined.

## 6 Falsifiable predictions and research implications

### 6.1 Empirically testable predictions

The following five predictions operationalize the central propositions of the biological state hypothesis into empirically testable and falsifiable statements. Each prediction specifies not only the expected finding if the hypothesis is correct but also the conditions under which it would be falsified—a requirement for any theoretical framework that aspires to scientific validity.

#### 6.1.1 Prediction 1: biological state as a predictor of adaptive capacity

Individuals with greater integrated physiological coherence—estimated through the minimum operational core of the biological state index assessed in basal, resting conditions (HRV, diurnal salivary cortisol profile, hs-CRP, sleep patterns and circadian regularity, VO_2_max, HOMA-IR, triglyceride:HDL ratio, serum *BDNF*, and epigenetic clocks)—will demonstrate greater adaptive capacity when confronted with environmental demands, reflected across two complementary dimensions: the psychological and cognitive dimension, expressed in cognitive flexibility under load, psychological resilience following adversity, and emotional regulation capacity under sustained stress; and the physiological and functional dimension, expressed in the speed and completeness of autonomic recovery following a standardized acute stressor, assessed across a pre-specified post-stressor recovery window, neuroendocrine recovery reflected in the cortisol return slope, metabolic recovery assessed through lactate clearance, and sustained functional capacity under demand approximated through VO_2_max in an effort protocol, handgrip dynamometry, or sit-to-stand performance. The trajectory of biological aging estimated longitudinally through DunedinPACE provides an additional expression of adaptive capacity over time. This prediction is falsifiable if individuals with multidomain biological state coherence—defined by simultaneously favorable values across the proposed minimum operational core—do not demonstrate significant differences in adaptive capacity outcomes across both dimensions compared with individuals with persistently compromised biological state, after controlling for chronological age, sex, and relevant confounding variables, in a prospective longitudinal study with repeated multidomain measurements. A null finding would specifically require that the BSI assessed in basal conditions fails to predict the dynamic functional expressions of adaptive capacity evaluated under standardized challenge conditions—constituting direct evidence against the central proposition of the biological state hypothesis.

#### 6.1.2 Prediction 2: biological state and biological aging

Individuals with greater acceleration of biological age estimated through second-generation epigenetic clocks—GrimAge or DunedinPACE—will demonstrate lower adaptive capacity and lower adaptive reserve, independently of chronological age and other relevant confounding factors. This prediction is falsifiable if accelerated epigenetic age does not add statistically significant predictive value for adaptive capacity outcomes once chronological age, sex, body mass index, and relevant clinical covariates are controlled in independent validation cohorts.

Second-generation epigenetic clocks function in this framework at two levels: as indicators of biological state when assessed at a single time point in basal conditions—reflecting the integrated accumulation of environmental and physiological influences on the organism’s epigenetic architecture—and as expressions of the trajectory of adaptive capacity when tracked longitudinally, reflecting whether the organism’s biological reserve is expanding, stable, or contracting in response to its accumulated life demands.

#### 6.1.3 Prediction 3: systemic regulation and biological plasticity

Persistent dysregulation of systemic regulation—reflected in reduced HRV, flattened diurnal cortisol slope, altered circadian rhythms, and elevated immune-inflammatory markers—will be prospectively associated with alterations in biological plasticity biomarkers, including reduced serum *BDNF*, accelerated epigenetic aging, and other indicators of impaired biological repair and remodeling capacity. This prediction establishes a specific interaction chain between the first and third layers of biological state that can be independently evaluated at each link and is falsifiable if persistent dysregulation of systemic regulation does not predict subsequent changes in biological plasticity indicators in longitudinal designs with a minimum follow-up of 12 months and repeated measurements at both layers.

#### 6.1.4 Prediction 4: incremental value of integrated biological state

Models integrating information from systemic regulation, metabolism, and biological plasticity simultaneously will provide greater explanatory and predictive capacity for adaptive capacity than models based on individual biomarkers or a single physiological domain. This prediction is falsifiable if a single-domain model achieves equivalent or superior predictive performance compared to the integrated multidomain model in independent external validation cohorts. Internal cross-validation is not sufficient to test this prediction—external validation in independent populations is required, and comparison must include simpler baseline models to rule out overfitting as an explanation for any observed advantage of the integrated model.

#### 6.1.5 Prediction 5: modifiability of biological state

In a randomized factorial trial with arms targeting each domain individually—systemic regulation, metabolism, and biological plasticity—as well as a combined multidomain arm simultaneously addressing all three layers through integrated strategies of aerobic and resistance exercise, precision nutrition, circadian synchronization, and mindfulness-based stress regulation, the combined multidomain intervention will produce greater-than-additive improvements in the biological state index and in adaptive capacity compared to the best-performing single-domain arm. This prediction is falsifiable if the combined multidomain intervention does not produce significantly greater improvements in the biological state index than the best single-domain arm in independent validation cohorts, or if the observed improvements are consistent with simple additivity rather than synergistic interaction among domains. A factorial design is explicitly required to test this prediction, as a pre–post multidomain study without single-domain comparison arms cannot distinguish synergistic effects from the sum of independent domain contributions.

## 7 Conclusion

The question that motivated this hypothesis—what determines how much adaptive capacity an organism retains at any given moment, and in which direction is that capacity trending?—remains without an integrative answer in the current literature. The biological state hypothesis proposes that adaptive capacity is an emergent functional property of integrated biological state—conceptually expressed as AC = g(BS)—whose amplitude is conditioned by the degree of physiological coherence among three interdependent layers: systemic regulation, metabolism, and biological plasticity. When these three layers maintain integrated organization, adaptive reserve is broad, and health is preserved. When persistent dysregulation erodes that coherence, adaptive reserve progressively diminishes—and it is this sustained narrowing, not any isolated system failure, that precedes and determines the emergence of chronic non-communicable disease and accelerated biological aging. Disease, in this framework, is not the starting point. It is the downstream consequence of a biological state that has lost its capacity to remain coherent under the demands of the environment.

This distinction carries a clinical implication that extends beyond conceptual elegance. If adaptive capacity deteriorates before overt pathology appears—as this framework proposes—then biological state can, in principle, be estimated, monitored, and intervened upon before the threshold of disease is crossed. The biological state hypothesis does not merely offer a new way of describing what goes wrong in chronic disease. It proposes a new temporal target for medicine: the integrated physiological organization of the organism, assessed longitudinally in basal conditions, before dysregulation becomes irreversible. Furthermore, it proposes a new functional target: the organism’s capacity to respond, recover, and reorganize its physiological coherence when confronted with environmental demands. This is the window that the framework seeks to open—not to replace existing diagnostic and therapeutic strategies, but to provide a physiological foundation for acting earlier, more precisely, and across the full system rather than within isolated domains.

The present proposal does not claim to offer a definitive explanation of biological adaptation. The interactions proposed among systemic regulation, metabolism, and biological plasticity have not yet been empirically validated as an integrated system, and the biological state index remains a hypothetical construct until prospectively demonstrated in independent populations. What this framework offers is something more foundational: a physiological question, formulated with sufficient precision to be answered through empirical research, and a conceptual architecture robust enough to guide that research systematically. Whether biological state assessed in basal conditions constitutes a measurable systemic constraint on adaptive capacity and whether its integrated organization predicts the organism’s functional behavior when confronted with environmental demands are questions that can now be put to the test. The principal contribution of the biological state hypothesis is not to have answered them. It is to have made them answerable.

## Data Availability

The original contributions presented in the study are included in the article/supplementary material. Further inquiries can be directed to the corresponding author.
